# Identifying Anticipated Events of Future Clinical Trials by Leveraging Data from the Placebo Arms of Completed Trials

**DOI:** 10.1007/s43441-020-00237-w

**Published:** 2020-11-09

**Authors:** Xiang-Lin Tan, David M. Kern, M. Soledad Cepeda

**Affiliations:** Janssen Research & Development, LLC, Titusville, NJ USA

**Keywords:** Anticipated events, Adverse events, Safety report, Clinical trials, Placebo, Bipolar disorder

## Abstract

**Background:**

An important component of a systematic strategy for safety surveillance is prospective identification of anticipated serious adverse events (SAEs). Developing a structured approach to identify anticipated events and estimating their incidence can help align the safety strategy and the safety surveillance efforts.

**Methods:**

We developed a novel approach to identify anticipated events for a hypothetical randomized, double-blind, controlled trial in subjects with bipolar disorder using the adverse events reported in the placebo arm of trials from the ClinicalTrials.gov database. We searched the ClinicalTrials.gov database for all trials on bipolar depression with similar inclusion/exclusion criteria and study duration as our hypothetical study. The frequencies of anticipated events in placebo arms were abstracted from each trial and 95% confidence intervals (CI) were calculated using the Clopper–Pearson method. Meta-analysis with a random effects model was performed to obtain a summary estimate and 95% CI for the events identified in more than one trial.

**Results:**

A total of 129 clinical trials were initially identified, and 18 were ultimately selected as they met all the selection criteria. There were 69 unique anticipated SAEs identified, and 13 out of 69 were reported in at least 2 clinical trials. The top 5 anticipated SAEs for our study were: (1) hospitalization, psychiatric symptom (3.57%); (2) suicidal behavior, overdose (3.57%), (3) cholecystitis (2.86%); (4) fall (2.86%); (5) road traffic accident, injury (2.86%).

**Conclusion:**

We successfully identified the anticipated events from registered trials that included a population similar to our trial. This method for identifying anticipated events could be applied to other disease areas.

**Electronic supplementary material:**

The online version of this article (10.1007/s43441-020-00237-w) contains supplementary material, which is available to authorized users.

## Background

Based on the 2012 Food and Drug Administration (FDA) *Guidance Safety Reporting Requirements for INDs (Investigational New Drug Applications) and BA/BE (Bioavailability/Bioequivalence) studies* [1], Sponsors are required to identify the anticipated events that it does not plan to report individually in an IND safety report. At the time of protocol development, these anticipated events should be included in the safety surveillance plan, together with a plan for monitoring the events.

Anticipated events are the adverse events (AEs) that the sponsor can foresee occurring with some frequency, independent of investigational drug exposure, in the general patient population under study, in patients with the disease under study, or both [2]. An important component of a systematic approach to safety surveillance is prospective identification of anticipated serious AEs (SAEs). The FDA has stated that individual case safety reports of such anticipated events are generally uninformative when reported as single events (i.e., without a comparison of the incidence of the event in treated and untreated subjects), and they do not contribute meaningfully to the developing safety profile of an investigational drug nor to subject protection [1, 2]. To reduce the number of uninformative individual reports, it is necessary to take a more structured approach to identify anticipated events and estimate frequency of occurrence.

Since the anticipated events are intended to be those events that are either due to underlying disease, concomitant therapy, or demographic characteristics [3], previous trials with a similar population can provide valuable assistance in the identification of anticipated events. The placebo arms of these analogous clinical trials are an ideal source to identify the anticipated events [4] as they by definition would exclude any events that may be truly attributable to the active medication. This formed the basis of our new approach to generate a list of anticipated events and their frequency using the AEs reported in the placebo arm of trials from the ClinicalTrials.gov results database.

In this proof of concept study, we sought to identify anticipated events from the registered clinical trials that are similar in population to a hypothetical clinical trial in bipolar depression. We used the Sherlock tool to identify the clinical trials that are similar to the hypothetical trial and extracted the information needed from the ClinicalTrials.gov database. This project provides an example of how to generate a list of anticipated events including serious and non-serious AEs using the ClinicalTrials.gov results database.

## Methods

### Hypothetical Study

We designed a hypothetical randomized double-blind controlled trial in subjects with bipolar disorder. The inclusion and exclusion criteria used reflect common research practice. Targeted participants are men or women between 18 and 65 years of age, with a *Diagnostic and Statistical Manual of Mental Disorders 5th edition* (DSM-5) diagnosis of bipolar disorder (types I and II), with or without mood stabilizing treatment. Participants must have experienced a major depressive episode (MDE), meeting the following criteria: (1) have a Hamilton Depression Rating Scale (HDRS_17_) total score ≥ 17; (2) have a minimum of 2 manic symptoms in the current MDE (3) have a Clinical Global Impression scale for bipolar disorder (CGI-BP) ≥ 3. Patients who have a primary DSM-5 diagnosis of borderline or anti-social personality disorder, schizoaffective disorder or rapid cycling bipolar disorder or meet the DSM-5 criteria for manic episode will be excluded. The total study duration for each participant will be approximately 14 weeks.

### Identification and Selection of Clinical Trials

The ClinicalTrials.gov is a registry and results database of both published and unpublished trials and contains information for privately and publicly funded clinical studies conducted worldwide. The ClinicalTrials.gov results database was launched in September 2008 to implement section 801 of the Food and Drug Administration Amendments Act of 2007 (FDAAA) (ClinicalTrials.gov) [5–7]. The law requires study sponsors to submit “basic results” for certain clinical trials, generally no later than 1 year after their completion date. The basic results include participant flow, baseline characteristics of the subjects included, outcome measures and statistical analyses, as well as information on observed AEs [8]. In the ClinicalTrials.gov results database, trial results are reported in a standard, tabular format which facilitates extraction of data with specialized software [6, 8]. To help facilitate the identification of appropriate trials, we developed Sherlock, a system that downloads the ClinicalTrials.gov database daily, and can be used to query the trials and create analytic files for further analyses [4, 9, 10].

**Fig. 1:**
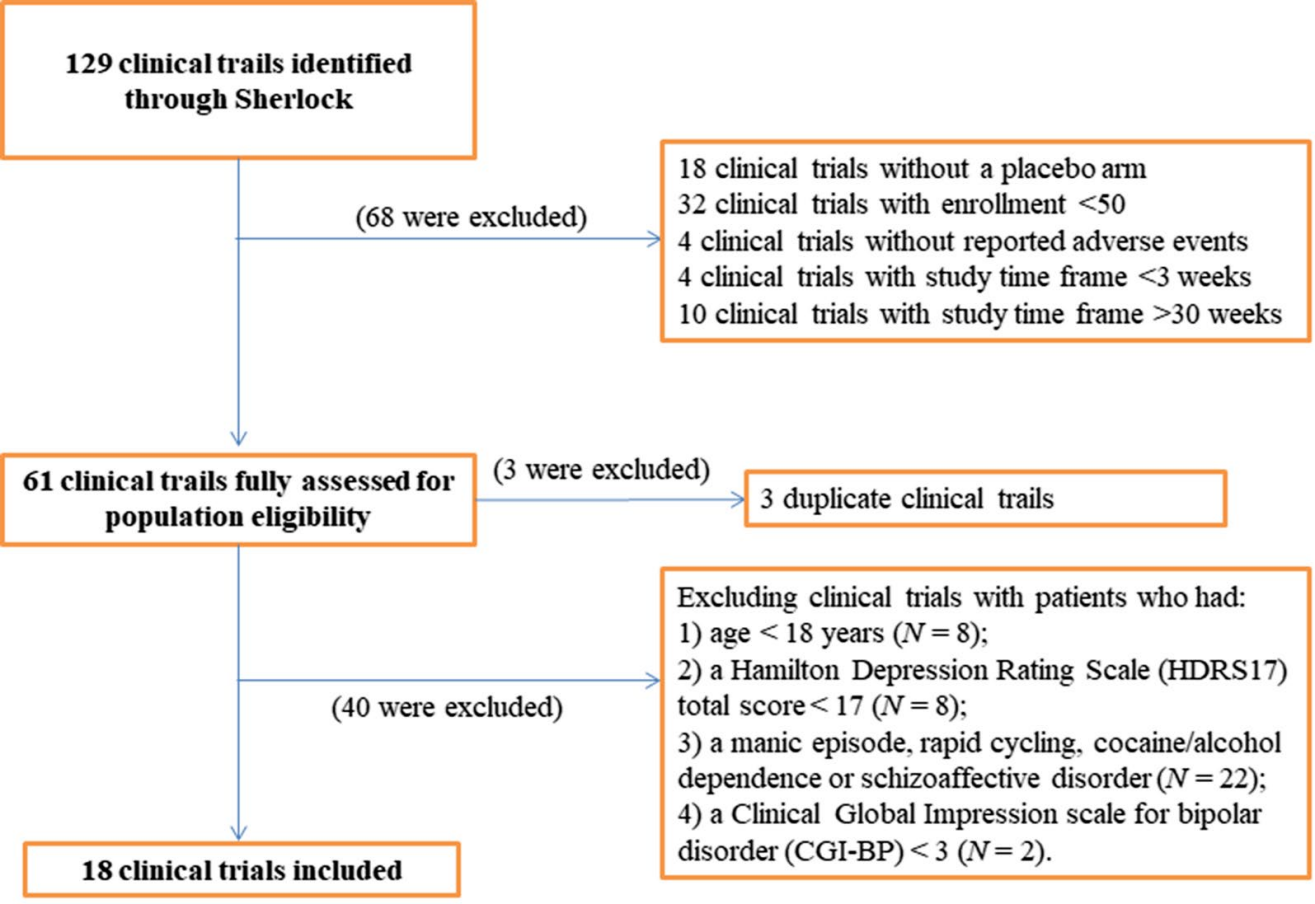
Flow scheme of selection of clinical trials for our hypothetical study

To identify potential clinical trials, we searched for clinical trials registered as of April 1, 2020, with the following search criteria: (1) trials with study results available; (2) condition terms with “bipolar disorders”, “bipolar affective disorder”, “bipolar I disorder”, “bipolar II disorder”, “depressed bipolar I disorder”, “mixed bipolar I disorder”, “moderate bipolar disorder”, or “severe bipolar disorder”; and (3) trials with placebo arm. We only included clinical trials with enrollment ≥ 50 subjects and with study-time frame ≥ 3 weeks and ≤ 30 weeks. Furthermore, we excluded clinical trials with patients who had: (1) Age < 18 years; (2) a HDRS_17_ total score < 17; (3) a manic episode, rapid cycling, cocaine/alcohol dependence or schizoaffective disorder; (4) a CGI-BP < 3 (Fig. 1). The authors reviewed the clinical trials that passed the first screening phase. In cases where there was disagreement as to the inclusion of a study, consensus was reached through discussion and by re-reviewing the clinicals trials’ study description and eligibility criteria.

### Identification of Anticipated Events

For the trials that met the inclusion/exclusion criteria, any AEs in the placebo arm reported in the Adverse Events section of the ClinicalTrials.gov results database were considered anticipated events. Anticipated events were further stratified according to the subcategories of the Adverse Event section: All-Cause Mortality, Serious Adverse Events and Other (not including serious) Adverse Events.

### Data Extraction

The following key trial information was extracted: clinical trial ID, study type, study phase, number of arms, total number of enrollment, study status, study time frame, study inclusion and exclusion criteria, group/arm title and description, type of the AEs (MedDRA preferred term), number of participants at risk, frequency threshold, number of reported AEs, and number of reported SAEs. A data collection spreadsheet was developed as an analytic file to facilitate data analyses.

### Statistical Analysis

For each selected clinical trial, the number of subjects who developed the AEs and SAEs, as well as the total number of subjects at risk in the placebo arm or the standard of care arm were extracted. For each anticipated event, incidence rates were calculated using the number of patients with the anticipated events and the total number of the subjects at risk. The frequency and the 95% confidence intervals (CI) were calculated using Clopper–Pearson method for a single proportion. For the AE and SAE identified in more than one trial, we performed a meta-analysis with a random effects model using R statistical software (https://www.R-project.org/) and the “DescTools”, “meta” and “metafor” packages to obtain a summary estimate and 95% confidence interval.

## Results

### Identification and Selection of Clinical Trials

There were 129 clinical trials initially identified (Fig. 1). Of these, 18 clinical trials were excluded because of the lack of a placebo arm. Then, we excluded 32 clinical trials with enrollment of less than 50 subjects or with a short study time frame (i.e. < 3 weeks) or a long study time frame (i.e. > 30 weeks). Four clinical trials which did not report AEs were also excluded. Finally, a total of 61 clinical trials were selected for the full assessment of the population eligibility.

After review in ClinicalTrials.gov, three duplicate clinical trials were excluded. We also excluded the clinical trials with pediatric patients (i.e. age at diagnosis < 18 years), and the clinical trials with the patients who had: (1) a HDRS_17_ total score < 17 (*N* = 8); (2) a manic episode, rapid cycling, cocaine/alcohol dependence or schizoaffective disorder (*N* = 22); 3) a CGI-BP < 3 (*N* = 2). Finally, 18 clinical trials were selected since they included a similar population to our clinical trial and met all the selection criteria (Table 1).

**Table 1:** Selected clinical trials which are similar to our hypothetical clinical trial

Clinical trial ID	No. of subjects		Key inclusion criteria	Follow-up time	Frequency threshold of reporting AE
	Total	Placebo			
NCT00186043	55	25	Patients with bipolar II disorder, YMRS > 12, MADRS > 14; Age: 18–65 years	8 weeks	0% for AE and SAE
NCT00377299	60	28	Patients with current major depressive episode (bipolar I, II) or major depressive disorder or amphetamine abuse or dependence; Age: 18–70 years	12 weeks	0% for SAE, 1% for other AE
NCT00402324	202	101	Patients with mixed episode of bipolar I disorder; previous manic or mixed episode; Age: 18–60 years	6 weeks	0% for SAE, 2% for other AE
NCT00481195	257	125	Patients with bipolar I disorder experiencing a major depressive episode; treated with lithium or valproic acid; Age: 18–65 years	8 weeks	0% for SAE, 3% for other AE
NCT00597896	50	24	Patients with bipolar I or II disorder; treated with standard mood stabilizer; Age: 18–65 years	8 weeks	0% for SAE, 5% for other AE
NCT00721955	314	105	patients with bipolar I disorder and acute agitation; Age: 18–65 years	30 days	0% for SAE, 5% for other AE
NCT00852202	234	77	Patients with bipolar I or II disorder without psychotic features, with current depressive episode; previous manic, hypomanic, mixed episode; HDRS ≥ 20; Age: 18–65 years	8 weeks	0% for SAE, 5% for other AE
NCT00868452	348	163	Patients with bipolar I disorder, with current depressive episode; previous manic or mixed episode; treated with lithium or divalproex; Age: 18–75 years	6 weeks	0% for SAE, 5% for other AE
NCT00868699	505	168	Patients with bipolar I disorder, with current depressive episode; previous manic or mixed episode; Age: 18–75 years	6 weeks	0% for SAE, 5% for other AE
NCT01133821	92	45	Patients with bipolar II disorder with a current major depressive episode; HDRS > 15; Age: 18–65 years	20 weeks	0% for SAE, 5% for other AE
NCT01256177	361	147	Patients with bipolar I or II disorder, most recent episode depressed; HDRS ≥ 20; Age: 18–65 years	8 weeks	0% for SAE, 5% for other AE
NCT01284517	356	171	Patients with bipolar I disorder, most recent episode depressed; previous manic or mixed episode; treated with lithium or divalproex; Age: 18–75 years	6 weeks	0% for SAE, 5% for other AE
NCT01409096	80	35	Patients with bipolar I, II or not otherwise specified (NOS) disorders, current major depressive episode; Age: 18–75 years	12 weeks	0% for AE and SAE
NCT01677182	535	184	Patients with bipolar I disorder, most recent depressed episode; treated with a mood stabilizer or antipsychotic; YMRS ≤ 10, MADRS ≥ 24, Age: 18–75 years	6 weeks	0% for SAE, 5% for other AE
NCT01725308	431	177	Patients with bipolar I or II disorder with a major depressive episode; HDRS ≥ 20; Age:20–64 years	8 weeks (period 1)	0% for SAE, 2% for other AE
NCT01986101	525	172	Patients with bipolar I disorder, most recent episode depressed without psychotic features; YMRS ≥ 20, Age: 18–74 years	6 weeks	0% for SAE, 5% for other AE
NCT02670538	493	165	Patients with bipolar I disorder, current major depressive episode, previous manic or mixed episode; HDRS ≥ 20; Age: 18–65 years	50 days	0% for SAE, 5% for other AE
NCT02989727	221	109	Patients with bipolar II disorder and currently depressed; HDRS ≥ 18; Age: > 18 years	8 weeks	0% for SAE, 5% for other AE

*HAMD or HDRS*, Hamilton depression rating scale 17-item, *MADRS* Montgomery Asberg depression rating scale, *YMRS* young mania rating scale scores, *AE* adverse events, *SAE*, Serious adverse events

### Types and Frequency of Anticipated Adverse Events

We identified a total of 154 unique anticipated AEs (MedDRA preferred term), and 91 out of 154 had at least one case from the placebo arm in at least one of the 18 clinical trials (Supplementary Table S1). Forty-one of 154 anticipated AEs had been reported in at least two clinical trials. A summary estimate and 95% confidence interval were calculated and reported for the AEs identified in more than one trial. The top 10 anticipated AEs for our studies were: (1) sedation complication (79.55%); (2) ill-defined disorder (20.0%); (3) headache (9.76%); (4) upper respiratory tract infection (8.42%); (5) nasopharyngitis (6.68%); (6) insomnia (6.0%); (7) nausea (5.69%); (8) dysgeusia (5.49%); (9) dizziness (5.45%); and (10) flatulence (5.19%) (Table 2, Supplementary Table S1).

**Table 2:** Top 25 anticipated events reported in the placebo arm of the 18 selected clinical trials

Anticipated event (preferred term)	No. of clinical trials	Proportion (%)	95% Confidence interval (CI)
Sedation complication	1	79.55	64.7–90.2
Ill-defined disorder	1	20.00	6.83–40.7
Headache	12	9.76	6.63–14.14
Upper respiratory tract infection	2	8.42	5.30–13.12
Nasopharyngitis	7	6.68	4.99–8.90
Insomnia	10	6.00	4.17–8.56
Nausea	11	5.69	3.86–8.31
Dysgeusia	2	5.49	2.98–9.91
Dizziness	8	5.45	3.48–8.45
Flatulence	1	5.19	1.43–12.77
Diarrhea	5	5.14	3.53–7.42
Somnolence	9	4.05	3.09–5.29
Toothache	1	4.00	1.31–9.09
Agitation	3	3.60	1.67–7.59
Hospitalization, psychiatric symptom	1	3.57	0.09–18.35
Suicidal behavior, overdose	1	3.57	0.09–18.35
Anxiety	2	3.39	1.28–8.68
Akathisia	8	3.36	2.17–5.15
Dry mouth	6	3.29	1.78–6.00
Cough	2	3.23	1.46–6.99
Initial insomnia	1	3.20	0.88–7.99
Vomiting	4	3.07	1.86–5.04
Sedation	5	3.04	1.90–4.83
Tremor	4	3.03	1.46–6.20
Constipation	3	3.01	1.63–5.47

### Types and Frequency of Anticipated Serious Adverse events

We also identified a total of 69 unique anticipated SAEs (MedDRA preferred term), and 36 out of 69 had at least one case from the placebo arm in at least one of the 18 clinical trials (Supplementary Table S2). Two deaths were reported in one clinical trial. Thirteen of 69 anticipated SAEs had been reported in at least 2 clinical trials. A summary estimate and 95% confidence interval were calculated and reported for the SAEs identified in more than one trial. The top 5 anticipated SAEs for our studies were: (1) hospitalization, psychiatric symptom (3.57%); (2) suicidal behavior, overdose (3.57%), (3) cholecystitis (2.86%); (4) fall (2.86%); and (5) road traffic accident, injury (2.86%) (Table 3, Supplementary Table S2).

**Table 3:** Top 25 anticipated serious adverse events reported in the placebo arm of the 18 selected clinical trials

Anticipated serious adverse event (preferred term)	No. of clinical trials	Proportion (%)	95% Confidence interval (CI)
Hospitalization, psychiatric symptom	1	3.57	0.09–18.35
Suicidal behavior, overdose	1	3.57	0.09–18.35
Cholecystitis	1	2.86	0.07–14.92
Fall	1	2.86	0.07–14.92
Road traffic accident, injury	1	2.86	0.07–14.92
Accident	2	1.92	0.27–12.43
Chest pain	2	1.60	0.40–6.17
Overdose	2	1.55	0.39–5.98
Death^a^	1	1.13	0.14–4.02
Asthenia	1	0.99	0.03–5.39
Hypoesthesia	1	0.99	0.03–5.39
Skin laceration	1	0.99	0.03–5.39
Agitation	1	0.92	0.02–5.01
Pneumonia	1	0.92	0.02–5.01
Bipolar disorder	2	0.83	0.21–3.24
Intentional self-injury	1	0.68	0.02–3.73
Peritonitis	1	0.68	0.02–3.73
Femur fracture	1	0.61	0.02–3.37
Lumbar vertebral fracture	1	0.61	0.02–3.33
Non-cardiac chest pain	1	0.61	0.02–3.33
Substance abuse	1	0.61	0.02–3.33
Bipolar I disorder	4	0.60	0.19–1.85
Duodenal ulcer	1	0.60	0.02–3.27
Abdominal pain	1	0.58	0.01–3.2
Acute myocardial infarction	1	0.58	0.01–3.2

^a^Two deaths were reported in one clinical trial

## Discussion

FDA safety reporting guidance indicates that sponsors should have a systematic approach to safety surveillance, including a process to pre-specify a list of anticipated SAEs to comply with the IND safety reporting requirements. In this study, we developed a novel approach to generate the list of anticipated SAEs using the placebo arm of studies with similar study designs and inclusion and exclusion criteria available in the clinicaltrials.gov results database. This list is presented here as a resource for sponsors of clinical trials to refer to while developing their pre-specified list of SAEs that are anticipated to occur in patients with bipolar disorder. This approach may serve as a model for identifying anticipated events in clinical trials investigating novel therapies for other diseases.

We were able to identify a large number of clinical trials that had similar inclusion and exclusion criteria, study design and duration of follow-up to our hypothetical study. This is crucial since characteristics of the study population and previous experience with a similar population are major factors in the identification of the anticipated events. The use of meta-analytic techniques allowed the researchers to incorporate potential heterogeneity of the studies.

Although the ClinicalTrials.gov results database has information of on publicly and privately funded clinical studies on a wide range of diseases and conditions, it does not contain information about all the clinical studies conducted in the USA because not all studies are required by law to post study results [11]. In addition, it is worth noting that the submission of AE information in ClinicalTrials.gov was required beginning in September 2009, and the results for the clinical trials before September 2009 are not available [5, 6].

A major benefit of the ClinicalTrials.gov reporting system, is that it contains results of “negative” studies, studies that did not differentiate from placebo, which are often not published [12]. In addition to including information on studies that are not published, ClinicalTrials.gov also provides more complete data than those that are published, and a reliance on published data may result in missing nearly two-thirds of AEs [13]. Of note in the present study is that many AEs and SAEs were reported in only one clinical trial. If these AEs are to be considered anticipated events one might expect them to occur in the majority of trials as they all include similar patient populations; however, AE reporting may be inconsistent across studies due to the high diversity in the number and type of possible adverse effects, variations in their definitions, methods of ascertainment and the size of the placebo arm. Additionally, in many studies there is a frequency threshold for reporting AEs, typically > 5% (Table 1), and thus we can't assume there were no events in studies where they aren’t reported. Because of this variability across studies, the prevalence estimates in the current study include only studies for which the AE was reported.

Other approaches that have been used to identify anticipated events is to utilize real world data [14]. We opted to use the AEs from placebo arms of similar studies because the inclusion and exclusion criteria can be mirrored, while it is more difficult to mirror the population in healthcare databases. Additionally, observational healthcare databases often do not have the depression scales needed to identify the subjects and the severity of the condition, which could change the rate of anticipated events such as suicidal ideation. Furthermore, the ascertainment of AEs in trials is completely different to how clinical events are recorded in healthcare databases, therefore, the AEs may be significantly underreported, which would make the rate of anticipated events obtained from those sources incomparable to the ones observed in a clinical trial. In addition, there is no real equivalent of a placebo patient in real-world databases, therefore, it is not possible to differentiate anticipated events from events caused by an active treatment.

## Conclusion

In this study, we have developed a novel approach for identifying anticipated events in clinical trials using the ClinicalTrials.gov results database. We showed that clinical trials with similar study designs and inclusion and exclusion criteria can be found, a list anticipated events can be obtained, and their rates calculated. This study may serve as a model for identifying anticipated events in clinical trials investigating novel therapies for other diseases.

## Electronic supplementary material

Below is the link to the electronic supplementary material.

Supplementary file1 (DOCX 28 kb)
