# Supplementary material for: Identifying Anticipated Events of Future Clinical Trials by Leveraging Data from the Placebo Arms of Completed Trials
Source: Ther Innov Regul Sci. 2020 Nov 9;55(2):454–61. doi: 10.1007/s43441-020-00237-w (PMC7864837; doi:10.1007/s43441-020-00237-w)
Supplement: Supplementary file 1 — Supplementary file1 (DOCX 28 kb) [file 43441_2020_237_MOESM1_ESM.docx]

**Supplementary Table 1.** Anticipated events (Preferred Terms) reported in the 18 selected clinical trials.

| **Anticipated event (Preferred Term)** | **No. of Clinical Trials** | **Proportion (%)** | **95% Confidence Interval (CI)** |
| --- | --- | --- | --- |
| Sedation complication | 1 | 79.55 | 64.7 - 90.2 |
| Ill-defined disorder | 1 | 20.00 | 6.83 - 40.7 |
| Headache | 12 | 9.76 | 6.63 - 14.14 |
| Upper respiratory tract infection | 2 | 8.42 | 5.30- 13.12 |
| Nasopharyngitis | 7 | 6.68 | 4.99 - 8.90 |
| Insomnia | 10 | 6.00 | 4.17 - 8.56 |
| Nausea | 11 | 5.69 | 3.86 - 8.31 |
| Dysgeusia | 2 | 5.49 | 2.98 - 9.91 |
| Dizziness | 8 | 5.45 | 3.48 - 8.45 |
| Flatulence | 1 | 5.19 | 1.43 - 12.77 |
| Diarrhoea | 5 | 5.14 | 3.53 - 7.42 |
| Somnolence | 9 | 4.05 | 3.09 - 5.29 |
| Toothache | 1 | 4.00 | 1.31 - 9.09 |
| Agitation | 3 | 3.60 | 1.67 - 7.59 |
| Hospitalisation, Psychiatric symptom | 1 | 3.57 | 0.09 - 18.35 |
| Suicidal behaviour, Overdose | 1 | 3.57 | 0.09 - 18.35 |
| Anxiety | 2 | 3.39 | 1.28 - 8.68 |
| Akathisia | 8 | 3.36 | 2.17 - 5.15 |
| Dry mouth | 6 | 3.29 | 1.78 - 6.00 |
| Cough | 2 | 3.23 | 1.46 - 6.99 |
| Initial insomnia | 1 | 3.20 | 0.88 - 7.99 |
| Vomiting | 4 | 3.07 | 1.86 - 5.04 |
| Sedation | 5 | 3.04 | 1.90 - 4.83 |
| Tremor | 4 | 3.03 | 1.46 - 6.20 |
| Constipation | 3 | 3.01 | 1.63 - 5.47 |
| Dyspepsia | 1 | 2.97 | 0.62 - 8.44 |
| Laryngeal pain | 1 | 2.97 | 0.62 - 8.44 |
| Cholecystitis | 1 | 2.86 | 0.07 - 14.92 |
| Fall | 1 | 2.86 | 0.07 - 14.92 |
| Road traffic accident, Injury | 1 | 2.86 | 0.07 - 14.92 |
| Blood prolactin increased | 1 | 2.82 | 0.92 - 6.47 |
| Thirst | 1 | 2.82 | 0.92 - 6.47 |
| Fatigue | 6 | 2.69 | 1.37 - 5.19 |
| Restlessness | 3 | 2.45 | 1.28 - 4.64 |
| Parkinsonism | 1 | 2.33 | 0.64 - 5.85 |
| Wound | 1 | 2.26 | 0.62 - 5.68 |
| Accident | 2 | 1.92 | 0.27 - 12.43 |
| Increased appetite | 2 | 1.87 | 0.40 - 8.25 |
| Abdominal pain upper | 2 | 1.80 | 0.75 - 4.25 |
| Chest pain | 2 | 1.60 | 0.40 - 6.17 |
| Overdose | 2 | 1.55 | 0.39 - 5.98 |
| Abdominal discomfort | 2 | 1.42 | 0.46 - 4.26 |
| Weight increased | 5 | 1.36 | 0.32 - 5.50 |
| Migraine | 1 | 1.30 | 0.03 - 7.02 |
| Death^a^ | 1 | 1.13 | 0.14 - 4.02 |
| Blood thyroid stimulating hormone increased | 1 | 1.13 | 0.14 - 4.02 |
| Blood triglycerides increased | 1 | 1.13 | 0.14 - 4.02 |
| Decreased appetite | 1 | 1.13 | 0.14 - 4.02 |
| Dizziness postural | 1 | 1.13 | 0.14 - 4.02 |
| Malaise | 1 | 1.13 | 0.14 - 4.02 |
| Palpitations | 1 | 1.13 | 0.14 - 4.02 |
| Back pain | 2 | 1.08 | 0.35 - 3.29 |
| Asthenia | 1 | 0.99 | 0.03 - 5.39 |
| Oedema peripheral | 1 | 0.99 | 0.03 - 5.39 |
| Skin laceration | 1 | 0.99 | 0.03 - 5.39 |
| Pneumonia | 1 | 0.92 | 0.02 - 5.01 |
| Bipolar disorder | 2 | 0.83 | 0.21 - 3.24 |
| Rhinorrhoea | 1 | 0.80 | 0.02 - 4.38 |
| Hypoaesthesia | 2 | 0.72 | 0.18 - 2.83 |
| Intentional self-injury | 1 | 0.68 | 0.02 - 3.73 |
| Peritonitis | 1 | 0.68 | 0.02 - 3.73 |
| Hypomania | 2 | 0.65 | 0.04 - 9.10 |
| Femur fracture | 1 | 0.61 | 0.02 - 3.37 |
| Lumbar vertebral fracture | 1 | 0.61 | 0.02 - 3.33 |
| Non-cardiac chest pain | 1 | 0.61 | 0.02 - 3.33 |
| Substance abuse | 1 | 0.61 | 0.02 - 3.33 |
| Bipolar I disorder | 4 | 0.60 | 0.19 - 1.85 |
| Duodenal ulcer | 1 | 0.60 | 0.02 - 3.27 |
| Abdominal pain | 1 | 0.58 | 0.01 - 3.2 |
| Acute myocardial infarction | 1 | 0.58 | 0.01 - 3.2 |
| Flank pain | 1 | 0.58 | 0.01 - 3.22 |
| Transient ischaemic attack | 1 | 0.58 | 0.01 - 3.22 |
| Suicidal ideation | 5 | 0.57 | 0.18 - 1.77 |
| Abortion spontaneous | 2 | 0.56 | 0.08 - 3.88 |
| Anaphylactic reaction | 1 | 0.56 | 0.01 - 3.11 |
| Aspartate aminotransferase increased | 1 | 0.56 | 0.01 - 3.11 |
| Blood creatine phosphokinase increased | 1 | 0.56 | 0.01 - 3.11 |
| Blood thyroid stimulating hormone decreased | 1 | 0.56 | 0.01 - 3.11 |
| Completed suicide | 1 | 0.56 | 0.01 - 3.11 |
| Contusion | 1 | 0.56 | 0.01 - 3.11 |
| Dental caries | 1 | 0.56 | 0.01 - 3.11 |
| Eczema | 1 | 0.56 | 0.01 - 3.11 |
| Gastroenteritis | 1 | 0.56 | 0.01 - 3.11 |
| Hyperprolactinaemia | 1 | 0.56 | 0.01 - 3.11 |
| Hyperventilation | 1 | 0.56 | 0.01 - 3.11 |
| Psychiatric symptom | 1 | 0.54 | 0.01 - 2.99 |
| Mania | 6 | 0.50 | 0.11 - 2.18 |
| Cellulitis | 2 | 0.34 | 0.05 - 2.38 |
| Rash | 3 | 0.31 | 0- 33.78 |
| Disease progression | 2 | 0.29 | 0.04 - 2.04 |
| Suicide attempt | 4 | 0.19 | 0 - 1.04 |
| Depression | 8 | 0.05 | 0 - 2.77 |
| Abdominal distension | 1 | 0^b^ | NC |
| Abortion | 1 | 0 | NC |
| Acute hepatic failure | 1 | 0 | NC |
| Alanine aminotransferase increased | 1 | 0 | NC |
| Alopecia | 1 | 0 | NC |
| Altered state of consciousness | 1 | 0 | NC |
| Anaphylactic shock | 1 | 0 | NC |
| Angioedema | 1 | 0 | NC |
| Appendicitis | 1 | 0 | NC |
| Arthralgia | 1 | 0 | NC |
| Asthma | 1 | 0 | NC |
| Atrial flutter | 1 | 0 | NC |
| Autonomic nervous system imbalance | 1 | 0 | NC |
| Blood glucose increased | 1 | 0 | NC |
| Blood pressure increased | 1 | 0 | NC |
| Bradykinesia | 1 | 0 | NC |
| Bronchitis | 1 | 0 | NC |
| Cardiac disorder, Palpitations | 1 | 0 | NC |
| Cystitis | 1 | 0 | NC |
| Depression suicidal | 1 | 0 | NC |
| Drug hypersensitivity, Subcutaneous abscess, Dermatitis allergic | 1 | 0 | NC |
| Drug use disorder, Alcohol use | 1 | 0 | NC |
| Dystonia | 1 | 0 | NC |
| Epididymal cyst | 1 | 0 | NC |
| Foot fracture | 1 | 0 | NC |
| Gamma-glutamyltransferase increased | 1 | 0 | NC |
| Head injury | 1 | 0 | NC |
| HIV infection | 1 | 0 | NC |
| Influenza | 1 | 0 | NC |
| Injury, Accident | 1 | 0 | NC |
| Intentional overdose | 1 | 0 | NC |
| Ligament sprain | 1 | 0 | NC |
| Loss of consciousness | 1 | 0 | NC |
| Musculoskeletal stiffness | 1 | 0 | NC |
| Myalgia | 1 | 0 | NC |
| Oral herpes | 1 | 0 | NC |
| Oropharyngeal pain | 1 | 0 | NC |
| Orthostatic hypotension | 1 | 0 | NC |
| Panic attack | 2 | 0 | NC |
| Panic attack, Sever's disease | 1 | 0 | NC |
| Pharyngitis | 1 | 0 | NC |
| Pyelonephritis | 1 | 0 | NC |
| Pyrexia | 1 | 0 | NC |
| Restless legs syndrome | 1 | 0 | NC |
| Retinal detachment | 1 | 0 | NC |
| Rhabdomyolysis | 1 | 0 | NC |
| Road traffic accident | 1 | 0 | NC |
| Sedation, Somnolence | 1 | 0 | NC |
| Sinusitis | 1 | 0 | NC |
| Small intestinal obstruction | 1 | 0 | NC |
| Spinal compression fracture | 1 | 0 | NC |
| Subcutaneous abscess | 1 | 0 | NC |
| Tachycardia | 1 | 0 | NC |
| Tendon rupture | 1 | 0 | NC |
| Tonsillitis | 1 | 0 | NC |
| Tooth abscess, Tooth infection | 1 | 0 | NC |
| Toxicity to various agents | 1 | 0 | NC |
| Tri-iodothyronine free increased | 1 | 0 | NC |
| Ulcerative keratitis | 1 | 0 | NC |
| Upper respiratory tract inflammation | 1 | 0 | NC |
| Urticaria | 1 | 0 | NC |
| Vertigo | 1 | 0 | NC |

^a^Two deaths were reported in one clinical trial.

^b^Note: For adverse events (AEs) where the proportion is zero, the AE was assessed but no subject in the placebo arm was affected, therefore, its 95% CI was not calculated (NC).

**Supplementary Table 2.** Anticipated serious adverse events (Preferred Terms) reported in the 18 selected clinical trials.

| **Anticipated serious adverse event (Preferred Term)** | **No. of Clinical Trials** | **Proportion (%)** | **95% Confidence Interval (CI)** |
| --- | --- | --- | --- |
| Hospitalisation, Psychiatric symptom | 1 | 3.57 | 0.09 - 18.35 |
| Suicidal behaviour, Overdose | 1 | 3.57 | 0.09 - 18.35 |
| Cholecystitis | 1 | 2.86 | 0.07 - 14.92 |
| Fall | 1 | 2.86 | 0.07 - 14.92 |
| Road traffic accident, Injury | 1 | 2.86 | 0.07 - 14.92 |
| Accident | 2 | 1.92 | 0.27 - 12.43 |
| Chest pain | 2 | 1.60 | 0.40 - 6.17 |
| Overdose | 2 | 1.55 | 0.39 - 5.98 |
| Death^a^ | 1 | 1.13 | 0.14 - 4.02 |
| Asthenia | 1 | 0.99 | 0.03 - 5.39 |
| Hypoaesthesia | 1 | 0.99 | 0.03 - 5.39 |
| Skin laceration | 1 | 0.99 | 0.03 - 5.39 |
| Agitation | 1 | 0.92 | 0.02 - 5.01 |
| Pneumonia | 1 | 0.92 | 0.02 - 5.01 |
| Bipolar disorder | 2 | 0.83 | 0.21 - 3.24 |
| Intentional self-injury | 1 | 0.68 | 0.02 - 3.73 |
| Peritonitis | 1 | 0.68 | 0.02 - 3.73 |
| Femur fracture | 1 | 0.61 | 0.02 - 3.37 |
| Lumbar vertebral fracture | 1 | 0.61 | 0.02 - 3.33 |
| Non-cardiac chest pain | 1 | 0.61 | 0.02 - 3.33 |
| Substance abuse | 1 | 0.61 | 0.02 - 3.33 |
| Bipolar I disorder | 4 | 0.60 | 0.19 - 1.85 |
| Duodenal ulcer | 1 | 0.60 | 0.02 - 3.27 |
| Abdominal pain | 1 | 0.58 | 0.01 - 3.2 |
| Acute myocardial infarction | 1 | 0.58 | 0.01 - 3.2 |
| Flank pain | 1 | 0.58 | 0.01 - 3.22 |
| Transient ischaemic attack | 1 | 0.58 | 0.01 - 3.22 |
| Suicidal ideation | 5 | 0.57 | 0.18 - 1.77 |
| Abortion spontaneous | 2 | 0.56 | 0.08 - 3.88 |
| Anaphylactic reaction | 1 | 0.56 | 0.01 - 3.11 |
| Completed suicide | 1 | 0.56 | 0.01 - 3.11 |
| Psychiatric symptom | 1 | 0.54 | 0.01 - 2.99 |
| Mania | 6 | 0.5 | 0.21 - 1.20 |
| Cellulitis | 2 | 0.34 | 0.05 - 2.38 |
| Disease progression | 2 | 0.29 | 0.04 - 2.04 |
| Suicide attempt | 4 | 0.19 | 0.03 - 1.31 |
| Depression | 7 | 0.09 | 0.01 - 0.67 |
| Abortion | 1 | 0^b^ | NC |
| Acute hepatic failure | 1 | 0 | NC |
| Altered state of consciousness | 1 | 0 | NC |
| Anaphylactic shock | 1 | 0 | NC |
| Angioedema | 1 | 0 | NC |
| Appendicitis | 1 | 0 | NC |
| Asthma | 1 | 0 | NC |
| Atrial flutter | 1 | 0 | NC |
| Depression suicidal | 1 | 0 | NC |
| Drug hypersensitivity, Subcutaneous abscess, Dermatitis allergic | 1 | 0 | NC |
| Epididymal cyst | 1 | 0 | NC |
| Foot fracture | 1 | 0 | NC |
| Head injury | 1 | 0 | NC |
| HIV infection | 1 | 0 | NC |
| Hypomania | 1 | 0 | NC |
| Injury, Accident | 1 | 0 | NC |
| Intentional overdose | 1 | 0 | NC |
| Loss of consciousness | 1 | 0 | NC |
| Panic attack | 2 | 0 | NC |
| Pyelonephritis | 1 | 0 | NC |
| Rash | 1 | 0 | NC |
| Restless legs syndrome | 1 | 0 | NC |
| Retinal detachment | 1 | 0 | NC |
| Rhabdomyolysis | 1 | 0 | NC |
| Road traffic accident | 1 | 0 | NC |
| Sinusitis | 1 | 0 | NC |
| Small intestinal obstruction | 1 | 0 | NC |
| Spinal compression fracture | 1 | 0 | NC |
| Subcutaneous abscess | 1 | 0 | NC |
| Tendon rupture | 1 | 0 | NC |
| Toxicity to various agents | 1 | 0 | NC |
| Ulcerative keratitis | 1 | 0 | NC |

^a^Two deaths were reported in one clinical trial.

^b^Note: For serious adverse events (SAEs) where the proportion is zero, the SAE was assessed but no subject in the placebo arm was affected, therefore, its 95% CI was not calculated (NC).
